# *In vivo* evaluation of apical third enlargements to twice and thrice larger than initial apical binding file in final treatment outcome

**DOI:** 10.6026/9732063002001116

**Published:** 2024-09-30

**Authors:** Kranti Rajesh Khadse, Rana K. Varghese, Malwika Sisodia, Naveen Kumar Gupta, Priyanka Damodhar Ippar, Anushri Arvind Uge

**Affiliations:** 1 Department of Conservative Dentistry and Endodontics, New Horizon Dental College and Research Institute, Sakri, Bilaspur, Chhattsigarh, India

**Keywords:** Root canal treatment, apical preparation, postoperative pain, periapical healing, Visual Analog Scale

## Abstract

Root canal treatment is a critical procedure in endodontics, aimed at eliminating microorganisms and pathological debris from the root canal system to prevent reinfection and ensure the health of the periradicular tissues. A total of 180 patients with asymptomatic apical periodontitis (PAI score >3) were included in this randomized clinical trial. Patients were divided into two main groups, each with three subgroups based on the biomechanical preparation of the canals using different file sizes and tapers. Biomechanical preparation was followed by intracanal medicament placement, and patients were recalled for obturation and final restoration. Postoperative pain was assessed using the Visual Analog Scale (VAS) at 6, 12, 24, 48 and 72 hours post-treatment. Periapical healing was evaluated using the Periapical Index (PAI) at 3,6 and 12 months post-treatment. While larger apical preparation sizes and tapers can enhance periapical healing, excessive enlargement beyond three sizes larger than the IABF does not significantly improve outcomes. Optimal root canal treatment should balance adequate cleaning and disinfection with the preservation of tooth structure.

## Background:

Root canal treatment refers to the process of using mechanical instruments to clean and shape the root canal system, removing any debris or infection and then filling it with a substance that does not react with the surrounding tissue, with the aim of preserving or restoring the health of the tissue around the root [1]. The main goal of this operation is to eradicate bacteria and pathological debris from the root canal system and avoid the occurrence of reinfection [2, 3]. Although these stages are important, the use of mechanical instruments along with irrigation is regarded as the most vital element in accomplishing this goal [4, 5]. Nevertheless, research has shown that the existing tools and methods used for cleaning and flushing the apical third of teeth are not entirely successful in removing both debris and germs. The challenge of eliminating bacterial debris from the apical third is due to the restricted canal space, intricate canal architecture, insufficient flushing of irrigants, and variability in the width of the root canal [6]. In order to achieve sufficient penetration of the irrigant and improve cleanliness, it has been recommended to increase the apical region [7]. The necessary degree of apical expansion, however, is a topic of contention. Advocates of greater apical preparations contend that it is the most efficient method for cleansing and sterilizing the canals. Increasing the size of the apical preparations improves the elimination of diseased dentin [8], enhances the flushing effect of irrigants in the apical area [9] and substantially decreases the amount of bacteria in the canal system [10]. Expanding the size of the canal, particularly using apical diameters such as #30 and #40, has been proposed as an efficient method for removing debris. Studies have shown that using preparation sizes of #45 and #60 to #80 may effectively decrease the amount of germs present during endodontic treatment [2, 8]. Yared and Dagher found that a #25 file may be just as effective as a #40 file in minimizing leftover microorganisms [11]. The conventional method is enlarging the root canal to a size three times greater than the initial apical binding file (FABF) [12]. Nevertheless, doubts have been raised about the efficacy of this method in guaranteeing consistent and adequate elimination of dentin from every part of the canal wall [13]. Studies on the morphometry of the apical portion of root canals indicate that it may be insufficient. While several studies suggest that expansion should be between six to eight times bigger than the FABF, other research indicates that canals in multi-rooted teeth may need to be enlarged to at least a #60 size in order to completely instrument the apical area [14]. This region is classified as a "critical zone" [5] because to the presence of unique anatomical features such as isthmuses, fins, ramifications and lateral canals. Several writers have stated that a greater apical preparation size, beyond the prior recommendations, is necessary to effectively disinfect the canal [6, 7]. Conversely, it is said that using a bigger preparation size causes unneeded dentin removal, which weakens the structure of the tooth. As a result, there is currently on-going discussion on the optimal size and taper of the preparation that may successfully disinfect the bottom part of the root canal while preserving dentin [8]. Several research have examined the impact of enlarging the apical size on reducing bacterial load [6], improving canal cleanliness [9], promoting healing of apical periodontitis after surgery and reducing postoperative discomfort [11] after endodontic therapy. Advocates of greater apical preparation sizes argue that it enables more effective penetration of irrigants and substantially decreases the presence of remaining bacteria in the root canal system [12]. Nevertheless, higher apical preparation sizes have several disadvantages, including the unwanted alteration of the original geometry of the canal, the weakening of the root, and procedural problems such as ledge development, transportation, and perforations [13]. The main benefits of limited apical enlargements are the preservation of tooth structure and the avoidance of obturating materials from being pushed out [14]. There is a suggestion that the last step of preparation should have a continuous taper with the shortest apical foramen feasible [15].

## Materials and Methods:

The research used a wide range of advanced instruments and equipment for performing endodontic treatments, including the Air rotor handpiece, Coltene rubber dam kit and a variety of endodontic files and burs from Dentsply Maillefer, Switzerland. The additional equipment included an apex finder, endomotor, ultrasonic endoactivator, and digital radiography devices manufactured by KODAK in Japan. A uniform strategy was used across all patient instances by using several irrigation solutions, intracanal medicaments, and materials for obturation and interim restorations. The research comprised patients who visited the Department of Conservative Dentistry & Endodontics at New Horizon Dental College & Research Institute in Bilaspur, Chhattisgarh. These patients were selected based on precise criteria for inclusion and exclusion. The research specifically targeted patients who had asymptomatic apical periodontitis with a PAI score of >3. Patients with systemic illnesses, pregnancy, or other contraindications to endodontic treatment were excluded from the trial. All participants provided informed permission after receiving a detailed explanation of the research concept, clinical procedures, and related hazards. The research had a total of 180 patients who were separated into two primary groups. Each group was then further divided into three subgroups depending on the biomechanical preparation of the canals. The treatment technique consisted of administering local anesthetic, preparing the access cavity, determining the working lengths using an apex finder and doing biomechanical preparation using different file systems with varying tapers. Postoperative pain was evaluated with the Visual Analog Scale (VAS) at various time points and the main outcome measures consisted of pain intensity and changes in periapical radiolucency during subsequent visits. After finishing the process of biomechanical preparation and placing medication within the canal, patients were asked to return for the last steps of filling and restoring the tooth. Radiographs were obtained to evaluate the healing of the periapical area and to compare the PAI scores at various time points throughout the follow-up period. Clinical success was assessed based on the lack of symptoms, including pain, soreness, sinus tract formation, or aberrant tooth movement, combined with the presence of normal periodontal probing depths.

## Results:

The inter-group comparison indicated that there were no notable disparities in the Periapical Index (PAI) values across the groups after 3 months. However, notable disparities were seen at 6 and 12 months among Group 1A, Group 1B and Group 1C, as well as between Group 2A, Group 2B and Group 2C (Table 1). No statistically significant differences were seen between Group 1A and Group 2A, as well as between Group 1C and Group 2C, at any of the time intervals examined (as shown in Table 3 and Table 4). However, there was a notable difference between Group 1B and Group 2B after 12 months, as seen in Table 3. Comparison among groups: Intra-group comparisons revealed diverse outcomes among various groups and time periods. Group 1A did not show any significant variations between the different time periods, suggesting a stable PAI score throughout the study period (Table 5). There was no significant difference detected in Group 1B between the 3 and 6-month intervals or between the 6 and 12-month intervals. However, a significant distinction was observed between the 3 and 12-month intervals. This indicates a gradual shift in the PAI score over an extended period of time, as seen in Table 5. Group 1C showed significant differences in the 3 and 12-month intervals, as well as the 6 and 12-month intervals, but not in the 3 and 6-month intervals. This indicates a more noticeable shift in the second half of the research period (Table 5). Within Group 2A, a notable disparity was seen between the 3 and 12-month periods, although no significant disparities were detected across the other intervals (Table 5). Groups 2B and 2C showed notable distinctions in the PAI scores between the 3 and 12-month intervals, as well as between the 6 and 12-month intervals. However, there were no significant differences between the 3 and 6-month intervals. This suggests that the changes in PAI scores were more noticeable after 6 months.

## Discussion:

This research was undertaken as a randomized prospective clinical trial to assess the influence of apical preparation size and taper on postoperative pain and the efficacy of primary endodontic treatment. The assessment of postoperative pain was conducted with the Visual Analog Scale (VAS), a well-recognized technique for measuring the degree of pain [4]. The VAS scale used in this investigation assigned a numerical value ranging from 1 (indicating no pain) to 4 (indicating severe pain) based on the measurement of distance on a 10-cm horizontal line [5]. There was no significant difference in postoperative pain detected across different groups at time intervals of 6, 12, 24, 48, and 72 hours, both within each group and between groups [6]. The research discovered that the expulsion of contaminated debris, irrigants, or intracanal medicaments into the periapical area may contribute to discomfort experienced after a surgical procedure [7]. The canal preparation in this trial was stopped 0.5-1 mm before reaching the radiographic apex. This might be the reason why there was no significant difference in the occurrence of postoperative discomfort between the different groups [8]. In addition, a few patients had little discomfort unrelated to the endodontic procedure, including dental sensitivity caused by clamp installation, prolonged mouth opening, or local anesthetic injection [9]. A significant association exists between the level of pain before and after surgery; individuals who have intense pain before the operation are more prone to have severe pain after the operation [10]. The lack of discomfort before surgery in this research may have influenced the lack of substantial variations in postoperative pain across the different groups [11]. Research has shown that using techniques such as irrigant activation, which involves the use of side-vented needles and ultrasonic irrigation devices like the Endoactivator, may effectively decrease postoperative pain [12]. Administering analgesia before to root canal treatment, known as pretreatment analgesia, may effectively decrease postoperative discomfort. This approach is especially beneficial for individuals who have a poor tolerance for pain [13]. Within 48 hours after therapy, pain significantly decreased in all groups, with 83% of patients reporting no pain and 17% reporting just light discomfort [14]. This discovery is consistent with the anticipation that minor discomfort is a frequent result after endodontic operation and usually diminishes during the first two days [15]. The second stage of this research evaluated the healing of the periapical region after endodontic treatment at 3, 6, and 12-month intervals. The Periapical Index (PAI) score system was used to quantify healing based on intraoral periapical radiographs. According to the findings, Group 2C, which used a binding file 3 size bigger than the initial apical binding file (IABF) with a 6% taper, had the best success rate. In this group, 93% of patients had PAI scores < 2 and showed no clinical indications or symptoms at the 12-month follow-up [1]. The success percentage of Group 1B, which had a 2% taper and was 2 sizes bigger than IABF, was much greater than that of Group 1A, which had a success rate of 20% [2].

## Conclusion:

Overall, the study demonstrated that while increasing the apical preparation size and taper can improve periapical healing, excessive enlargement beyond 3 sizes larger than the IABF does not significantly increase the success rate.

## Figures and Tables

**Table 1:** Inter group PAI comparison

**Time Interval**	**PAI (Mean ± SD)**	**Group 1A**	**Group 1B**	**Group 1C**	**F Value (ANOVA One-way)**	**Significant Groups (P<0.05)**
At 3 months		3.9 ± 0.71	3.83 ± 0.7	3.53 ± 0.68	2.35 (p>0.05)	-
At 6 months		3.57 ± 0.5	3.17 ± 0.65	2.57 ± 0.68	20.09 (p<0.01)	1A vs 1B, 1B vs 1C, 1A vs 1C
At 12 months		3.23 ± 0.57	2.33 ± 0.71	1.77 ± 0.73	36.23 (p<0.01)	1A vs 1B, 1B vs 1C, 1A vs 1C
**Time Interval**	**PAI (Mean ± SD)**	**Group 2A**	**Group 2B**	**Group 2C**	**F Value (ANOVA One-way)**	**Significant Groups (P<0.05)**
At 3 months		3.9 ± 0.8	3.8 ± 0.58	3.4 ± 0.66	0.44 (p>0.05)	-
At 6 months		3.57 ± 0.73	2.8 ± 0.61	2.5 ± 0.73	12.87 (p<0.01)	2B vs 2C, 2A vs 2C
At 12 months		3.07 ± 0.74	1.57 ± 0.68	1.4 ± 0.63	22.5 (p<0.01)	2A vs 2B, 2A vs 2C

**Table 2:** Inter group comparison between Group 1A and Group 2A

**Time Interval**	**PAI (Mean ± SD)**	**Group 1A**	**Group 2A**	**P Value**
At 3 months		3.9 ± 0.71	3.9 ± 0.8	0.99 NS
At 6 months		3.57 ± 0.5	3.57 ± 0.73	0.99 NS
At 12 months		3.23 ± 0.57	3.07 ± 0.74	0.33 NS

**Table 3:** Inter group comparison between Group 1B and Group 2B

**Time Interval**	**PAI (Mean ± SD)**	**Group 1B**	**Group 2A**	**P Value**
At 3 months		3.83± 0.7	3.8± 0.58	0.96 NS
At 6 months		3.17± 0.65	2.8± 0.61	0.55 NS
At 12 months		2.33± 0.71	1.57± 0.68	0.02 S

**Table 4:** Inter group comparison Between Group 1C and Group 2C

**Time Interval**	**PAI (Mean ± SD)**	**Group 1C**	**Group 2A**	**P Value**
At 3 months		3.53± 0.68	3.4± 0.66	0.13 NS
At 6 months		2.57± 0.68	2.5± 0.73	0.27 NS
At 12 months		1.77± 0.73	1.4± 0.63	0.25 NS

**Table 5:** Intra Group PAI Comparison

Group	Time Interval	PAI (Mean± SD)	Time Points	P Value (NS = Not Significant, HS = Highly Significant)
1A	At 3 months	3.9± 0.71	3 months vs 6 months	P > 0.05 NS
	At 6 months	3.57± 0.5	3 months vs 12 months	P > 0.05 NS
	At 12 months	3.23± 0.57	6 months vs 12 months	P > 0.05 NS
1B	At 3 months	3.83± 0.7	3 months vs 6 months	P > 0.05 NS
	At 6 months	3.17± 0.65	3 months vs 12 months	P < 0.01 HS
	At 12 months	2.33± 0.71	6 months vs 12 months	P > 0.05 NS
1C	At 3 months	3.53± 0.68	3 months vs 6 months	P > 0.05 NS
	At 6 months	2.57± 0.68	3 months vs 12 months	P < 0.01 HS
	At 12 months	1.77± 0.73	6 months vs 12 months	P < 0.01 HS
2A	At 3 months	3.9± 0.8	3 months vs 6 months	P > 0.05 NS
	At 6 months	3.57± 0.73	3 months vs 12 months	P < 0.01 HS
	At 12 months	3.07± 0.74	6 months vs 12 months	P > 0.05 NS
2B	At 3 months	3.8± 0.58	3 months vs 6 months	P > 0.05 NS
	At 6 months	2.8± 0.61	3 months vs 12 months	P < 0.01 HS
	At 12 months	1.57± 0.68	6 months vs 12 months	P < 0.01 HS
2C	At 3 months	3.4± 0.66	3 months vs 6 months	P > 0.05 NS
	At 6 months	2.77± 0.73	3 months vs 12 months	P < 0.01 HS
	At 12 months	1.4± 0.63	6 months vs 12 months	P < 0.01 HS
